# 

**Published:** 1852-01

**Authors:** 


### CONTENTS

OF THE

### MEDICAL EXAMINER.

NEW SERIES—NO. LXXXV.—JANUARY, 1852.

### ORIGINAL COMMUNICATIONS.

Annual Report of the Committee on Public Hygiene, read before the Northern Medical Association of Philadelphia, Nov. 20th, 1851, by the Chairman, Wilson Jewell, M. D., and ordered to be published, - - - - - - 1

Thoughts on the Study of Diseases, with reference to Geology. Communicated to the Medical Society of Berks County, Nov. 4th, 1851, by J. B. Hiester, M. D., - - - - 19

A Case of Spasmodic Asthma. Reported by H. A. Swasey, M. D., of Mississippi, - - - - - - - 23

Case of Bed-sore successfully treated by a Galvanic Current. By W. S. W. Ruschenberger, M. D., Surgeon, U. S. Navy, - 25

Extracts from a Lecture "On the present position in Europe of some of the most interesting and important points of Modern Surgery," recently delivered as an Introductory Discourse. By Thomas D. Mütter, M. D., Professor of Surgery in Jefferson Medical College, Philadelphia, - - - - - 28

### BIBLIOGRAPHICAL NOTICES.

Manual of Physiological Chemistry. By Prof. Dr. C. G. Lehmann, 35

Manual of Physiological and Pathological Chemistry, after the most recent sources. Prepared by Dr. A. Moser and Dr. J. C. Strahl, - - - - - - - 35

Physiology of the Interchange of Matter in Plants and Animals. A Manual for Naturalists, Agriculturists, and Physicians. By Dr. Jac. Moleschott, &c. &c., - - - - 35

Familiar Letters on Chemistry in its relations to Physiology, Dietetics, Agriculture, Commerce, and Political Economy. By Justus Von Liebig, - - - - - - 35

The Elements of Materia Medica and Therapeutics. By Jonathan Pereira, M. D., F. R. S., L. S. Third American Edition, enlarged and improved by the Author. Edited by Joseph Carson, M. D., Professor of Materia Medica and Pharmacy in the University of Pennsylvania, etc., - - - - 44

A Complete Treatise on Midwifery; or the Theory and Practice of Tokology; including the Diseases of Pregnancy, Labor, and the Puerperal state. By Alf. A. L. M. Velpeau, M. D. Translated from the French by Charles D. Meigs, M. D., Professor of Midwifery in the Jefferson Medical College, &c. Fourth American, with the additions from the last French Edition, by Wm. Byrd Page, M. D., Lecturer on Obstetrics in the Philadelphia Medical Institute, &c., - - - - 45

An Analytical Compendium of the various branches of Medical Science, for the use and examination of Students. By John Neill, M. D., Demonstrator of Anatomy in the University of Pennsylvania, etc., and Francis Gurney Smith, M. D., Lecturer on Physiology in the Philadelphia Association for Medical Instruction, etc., - - - - - - 48

Lectures on Scarlet Fever. By Caspar Morris, M. D., Late Lecturer on Practical Medicine in the Philadelphia Medical Institute, Fellow of the College of Physicians of Philadelphia, &c. &c., 48

### EDITORIAL.

Our Journal, - - - - - - - - 49
Delegates from the Medical Society of Delaware, - - - 49
Monument to Dr. Jenner, - - - - - - - 50
Lactation in an Infant, - - - - - - 50
Death of Dr. Leiper, - - - - - - - - 50
Report of a Joint Committee of the Philadelphia County Medical Society and the Philadelphia College of Pharmacy, relative to Physicians' Prescriptions, - - - - - - 50

### MEDICAL NEWS.

University of New York, - - - - - - 55
Veterinary Journal, - - - - - - - - 56
Primary Medical School, - - - - - - - 56
Cost of the Doctorate in Paris, - - - - - 56
Action of Water on Lead Pipes, - - - - - 56
Committee on the Radical Cure of Reducible Hernia, - 58
Epidemic Jaundice, - - - - - - - - 58
George Ord, Esq., - - - - - - - 58
Death of Dr. R. L. Hooper, - - - - - - 58
Testimonial to Dr. Lever, - - - - - - 59
Deaths from Chloroform, - - - - - - 59

### RECORD OF MEDICAL SCIENCE.

### PATHOLOGY AND PRACTICE OF MEDICINE.

Case of Colica Pictonum, from the Medical Employment of Acetate of Lead. With Remarks. By L. S. Joynes, M. D., of Accomack, - - - - - - - 60

### SURGERY.

Report of a Successful Case of Trephining for Compression of the Brain. By J. W. H. Trugien, of Portsmouth, Va., - - 64

